# c-Abl tyrosine kinase down-regulation as target for memory improvement in Alzheimer’s disease

**DOI:** 10.3389/fnagi.2023.1180987

**Published:** 2023-06-05

**Authors:** Rilda León, Daniela A. Gutiérrez, Claudio Pinto, Cristian Morales, Catalina de la Fuente, Cristóbal Riquelme, Bastián I. Cortés, Adrián González-Martin, David Chamorro, Nelson Espinosa, Pablo Fuentealba, Gonzalo I. Cancino, Silvana Zanlungo, Andrés E. Dulcey, Juan J. Marugan, Alejandra Álvarez Rojas

**Affiliations:** ^1^Cell Signaling Laboratory, Department of Cellular and Molecular Biology, Biological Sciences Faculty, Millennium Institute on Immunology and Immunotherapy, Pontificia Universidad Católica de Chile, Santiago, Chile; ^2^Laboratory for Brain-Machine Interfaces and Neuromodulation, Facultad de Ingeniería, Instituto de Ingeniería Biológica y Médica, Pontificia Universidad Católica de Chile, Santiago, Chile; ^3^Laboratory of Neural Circuits, Department of Psychiatry, Neuroscience Interdisciplinary Centre, Pontificia Universidad Católica de Chile, Santiago, Chile; ^4^Department of Cellular and Molecular Biology, Biological Sciences Faculty, Pontificia Universidad Católica de Chile, Santiago, Chile; ^5^Department of Gastroenterology, Faculty of Medicine, Pontificia Universidad Catolica de Chile, Santiago, Chile; ^6^Early Translation Branch, National Center for Advancing Translational Sciences (NCATS), NIH, Rockville, MD, United States

**Keywords:** c-Abl inhibitors, tyrosine kinases, Alzheimer’s disease, memory, Hippocampi

## Abstract

**Background:**

Growing evidence suggests that the non-receptor tyrosine kinase, c-Abl, plays a significant role in the pathogenesis of Alzheimer’s disease (AD). Here, we analyzed the effect of c-Abl on the cognitive performance decline of APPSwe/PSEN1ΔE9 (APP/PS1) mouse model for AD.

**Methods:**

We used the conditional genetic ablation of c-Abl in the brain (c-Abl-KO) and pharmacological treatment with neurotinib, a novel allosteric c-Abl inhibitor with high brain penetrance, imbued in rodent’s chow.

**Results:**

We found that APP/PS1/c-Abl-KO mice and APP/PS1 neurotinib-fed mice had improved performance in hippocampus-dependent tasks. In the object location and Barnes-maze tests, they recognized the displaced object and learned the location of the escape hole faster than APP/PS1 mice. Also, APP/PS1 neurotinib-fed mice required fewer trials to reach the learning criterion in the memory flexibility test. Accordingly, c-Abl absence and inhibition caused fewer amyloid plaques, reduced astrogliosis, and preserved neurons in the hippocampus.

**Discussion:**

Our results further validate c-Abl as a target for AD, and the neurotinib, a novel c-Abl inhibitor, as a suitable preclinical candidate for AD therapies.

## 1. Introduction

Alzheimer’s disease (AD) is a neurodegenerative disorder characterized by cognitive decline primarily affecting memory (Lane et al., 2018; Knopman et al., 2021) being the leading form of dementia with a contribution in 60–70% of worldwide cases.

AD patient’s brains have a loss of neuronal populations in regions related to memory and cognition, such as the hippocampus, amygdala, and frontal regions (Gomez-Isla et al., 2008; Viola and Klein, 2015), and are characterized by extracellular aggregates of the Amyloid-β peptide (Aβ) and intracellular neurofibrillary tangles aggregates form by hyper-phosphorylated cytoskeletal Tau protein (Walsh and Selkoe, 2004). The earliest changes in AD are associated with the accumulation of oligomeric forms of the Aβ peptide (AβOs), leading to progressive reduction in connectivity and loss of plasticity followed by synaptic loss, neurotransmitter depletion, cytoskeletal alterations, abnormal protein phosphorylation, and finally, neuronal death (Davies et al., 1987; Hsia et al., 1999; Atwood and Bowen, 2015). AD typically presents with prominent amnestic cognitive impairment and short-term memory difficulty, but impairment in expressive speech, visuospatial processing, and executive functions also occurs. Most AD cases are not dominantly inherited and have a complex relationship to genetics.

The c-Abl protein has been involved in the pathogenesis of many neurodegenerative diseases. It belongs to the ABL family of non-receptor tyrosine kinases that comprises c-Abl (ABL1) and Arg (ABL2). c-Abl can regulate the actin cytoskeleton in dendrites (Jones et al., 2004; Chandía-Cristi et al., 2023) and interact with p73 to promote neuronal death (Wang, 2014; Khatri et al., 2016; Gutiérrez et al., 2023). Neurons treated with AβOs or fibrils show c-Abl activation, and this activation correlates with AD pathology in patients’ brains (Alvarez et al., 2004; Jing et al., 2009). Inhibition of c-Abl with imatinib (ATP binding site orthosteric inhibitor) prevented tau phosphorylation, the blockade of LTP induction, dendritic spine reduction, and neuronal apoptosis caused by Aβ (Cancino et al., 2011; Vargas et al., 2014). c-Abl inhibition increased the expression of several synaptic genes in AD models through HDAC2 stabilization (González-Zuñiga et al., 2014), while c-Abl null neurons showed reduced susceptibility to synaptic contacts elimination caused by AβOs (Gutiérrez et al., 2019). González-Martín and colleagues have published the effect of c-Abl ablation in cognitive training as c-Abl KO mice had improved performance when subjected to learning tasks such as the Morris water maze test (González-Martin et al., 2021).

Chronic intraperitoneal administration of imatinib in the AD mouse model APPSwe/PSEN1ΔE9 (APP/PS1) reduced behavioral deficits and decreased AβOs accumulation in the APP/PS1 mice brain and blood (Cancino et al., 2008; Estrada et al., 2016). Therefore, c-Abl inhibition can modulate mice’s cognitive performance. However, one of the limitations of using current FDA-approved c-Abl inhibitors is that they have a poor blood–brain barrier (BBB) permeability and target other kinases (Greuber et al., 2013; Aghel et al., 2017; Zukas and Schiff, 2018). In AD, a nilotinib (c-Abl orthosteric inhibitor) trial of 37 patients using the maximal daily tolerated dose of 300 mg showed that after 6–12 months of treatment, the group had reduced Aβ_40_ and Aβ_42_ cerebrospinal fluid levels compared to the placebo group, displaying also reduced Tau phosphorylation (Turner et al., 2020). Although the pathological biomarkers were reduced, the severity and progression of cognitive impairment did not change. However, patients only reached 2–4 nM CSF levels of nilotinib, 10–20 times less than its IC50 against cAbl. These low levels of exposure in CNS might be the cause behind the discrepancies regarding observed cognitive improvement in animal models and human clinical trials. Recently, our group has designed and characterized neurotinib, a new c-Abl allosteric inhibitor binding at the myristoyl pocket of the kinase with excellent BBB permeability and brain pharmacokinetics, and showed that neurotinib decreases cognitive decline in Niemann-Pick A mice (Marin et al., 2022).

Here, we have ultimately confirmed the role of c-Abl in the pathogenesis of AD by generating a new AD transgenic mice strain with genetic ablation of c-Abl in neurons. The APPswe/PSEN1ΔE9/c-Abl-KO (APP/PS1/c-Abl KO) mice had improved performance in hippocampus-dependent tasks than the APP/PS1 mice. Interestingly, APP/PS1/c-Abl KO mice null for c-Abl or APP/PS1 mice fed with neurotinib showed better performance in hippocampus-dependent tasks and reduced brain amyloid burden.

Altogether, our results suggest that c-Abl exerts an important role in the loss of hippocampal-dependent memory in AD. Moreover, we show that c-Abl is a relevant player in AD pathology and that its absence is beneficial for AD, strengthening the use of c-Abl inhibitors as potential disease-modifying drugs for this neurodegenerative disorder and validating neurotinib as a suitable preclinical development candidate for the treatment of AD.

## 2. Materials and methods

### 2.1. Animals

Animals were housed in a temperature and humidity-controlled room (22 ± 2°C) with food and water *ad libitum*. c-Abl-KO mice (Abl^loxP/loxP^/Nestin-CRE) mice were bred from Abl^loxP/loxP^ (these floxed mutant mice possess loxP sites flanking exon 5 of the *Abl1* gene) mice with Nestin-CRE+ mice (Jackson Laboratory), resulting in a brain-specific c-Abl conditional knock-out mouse. This strain originated and was maintained on a mixed B6.129S4, C57BL/6 background and did not display any gross physical or behavioral abnormalities. The APPswe/PSEN1ΔE9 mice were purchased from Jackson Laboratory (Bar Harbor, ME, United States), number 34829-JAX. The trade name B6;C3-Tg(APPswe,PSEN1dE9)85Dbo/Mmjax and genetic background is C57BL/6;C3H (Finnie et al., 2017).

To obtain the four genotypes used in this work, we initially crossed APPswe/PSEN1ΔE9 mice with Abl^loxP/loxP^ mice, resulting in APPswe/PSEN1ΔE9/Abl^loxP/loxP^ mice. Then the APPswe/PSEN1ΔE9/Abl^loxP/loxP^ mice were bred with Abl^loxP/loxP^/Nestin-CRE mice, resulting in the four genotypes of interest: APPswe/PSEN1ΔE9/Abl^loxP/loxP^ mice (APP/PS), APPswe/PSEN1ΔE9/Abl^loxP/loxP^/Nestin-CRE mice (APP/PS1/Abl-KO), Abl^loxP/loxP^ mice (WT), and Abl^loxP/loxP^/Nestin-CRE mice (c-Abl-KO). PCR was used to confirm the genotypes of all animals. The animal study protocol #170616008 was approved by the Bioethics and Care of Laboratory Animals Committee of the Pontificia Universidad Católica de Chile and CIBEM.

### 2.2. Neurotinib diet administration

16-month-old WT and APP/PS1 mice began being fed a control diet or diet containing 67 ppm of neurotinib. At 20-month-old the animals started sequential cognitive tests with resting periods between tests, during which the respective diets were maintained until mice were sacrificed and tissues collected for immunofluorescence. The mice were fed with control or neurotinib (patent number WO2019/173761 A1) containing diet *ad libitum.* The rodent diet was manufactured by Envigo/Teklad (Madison, Wisconsin) by incorporation of neurotinib ppm at 67 ppm into the NIH-31 Open Formula Mouse/Rat Sterilizabile Diet (7017), followed by irradiation handling of the final product.

### 2.3. Behavioral testing

Before behavioral testing, all animals were familiarized with the testing room, and the tests were performed from less anxiogenic to most anxiogenic (Open Field, Novel Object Recognition, Object Location Memory, Barnes maze, Memory Flexibility, Morris water maze), between 3 days from each other to let the animals rest.

#### 2.3.1. Open field, novel object recognition, and object location memory tests

**Figure 1:**
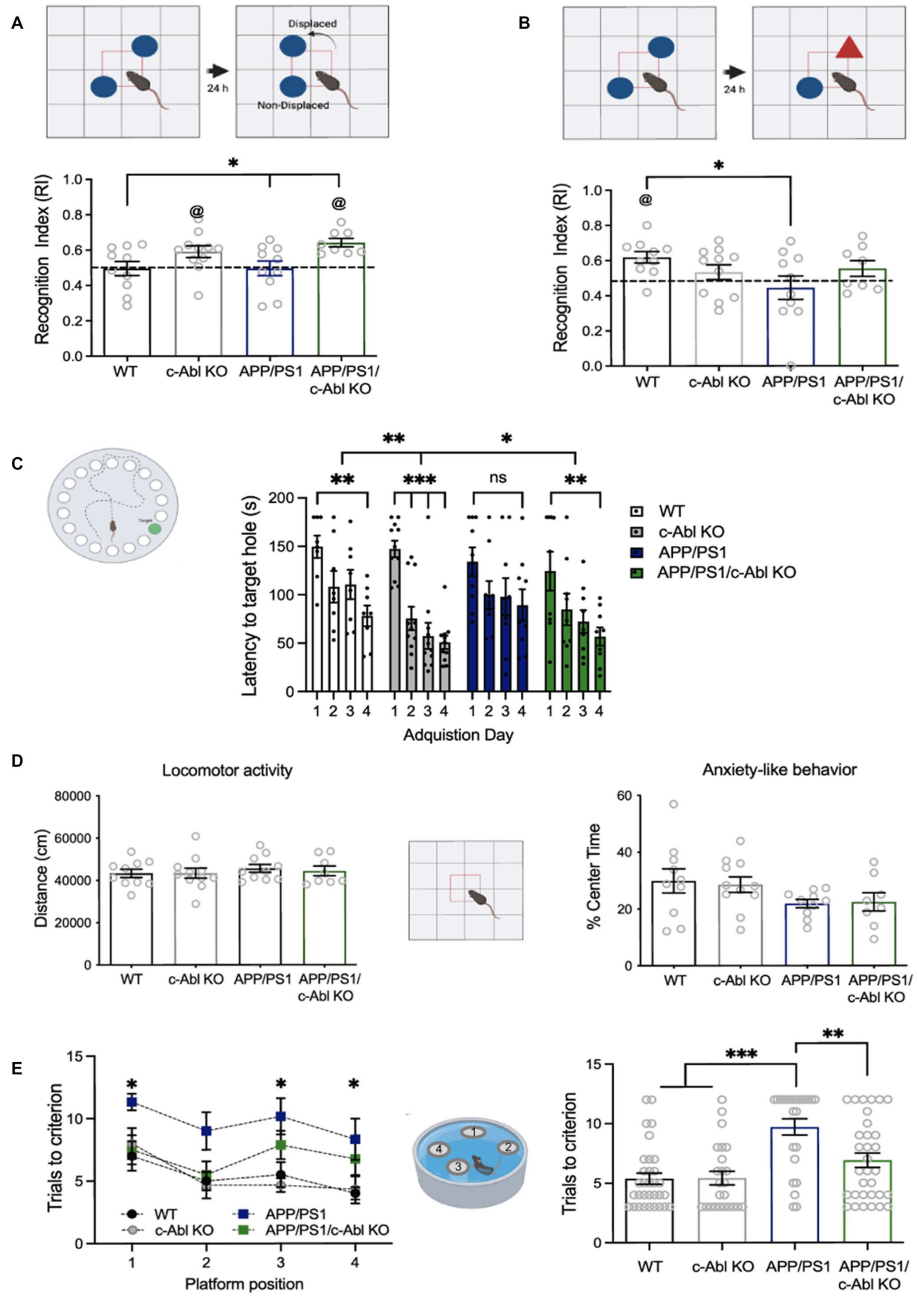
c-Abl absence improves behavioral performance in hippocampus-dependent tasks. 10-month-old wild-type (WT), c-Abl conditional knock-out mice (c-Abl KO), and AD mice either WT for c-Abl (APP/PS1) or knock-out for c-Abl (APP/PS1/c-Abl KO) were sequentially subjected to learning tasks as indicated by each cartoon. **(A)** Object Location Memory test. The graph shows the time spent on the relocated object compared to the total exploration time. APP/PS1 vs. APP/PS1/c-Abl KO *p* = 0.0506 Tukey’s *post-hoc* test. Two-way ANOVA p** = 0.0024 genotype effect, Tukey’s multiple comparison test @ *p* < 0.05 vs. chance level (0.5). **(B)** Novel Object Recognition test. Graph shows the time spent smelling the new object, compared with total exploration time. APP/PS1 vs. APP/PS1/c-Abl KO *p* = 0.4407 Tukey’s *post-hoc* multiple comparison test. Two-way ANOVA *p* = 0.0552 genotype (Continued)FIGURE 1 (Continued)and APP condition effect; @ *p* < 0.05 vs. chance level (0.5). **(C)** Graph shows the time spent finding the target hole each day of the Barnes maze test. Two-way ANOVA p** = 0.0054 genotype effect, p*** < 0.0001 acquisition day effect, Tukey’s *post-hoc* multiple comparison test. **(D)** Open Field tests. In each case, the bar shows the percent of time spent exploring the center zone (Anxiety-like behavior, *p* = 0.7625) and the distance traveled during the whole exploratory time (basal locomotor activity, *p* = 0.7690). Two-way ANOVA, Tukey’s *post-hoc* multiple comparison test. Same *n* = 10 WT, 10 APP/PS1, 8 APP/PS1/c-Abl KO, and 11 c-Abl KO animals on each test. **(E)** Memory flexibility test of 10-month-old mice. Left: graph show the number of mice attempts to find the platform in each position at different training days. Asterisks indicate WT vs. APP/PS1 significance through training days (1st day *p* = 0.0419, 3rd day *p* = 0.0242, 4th day *p* = 0.0419; APP vs. APP/PS1/c-Abl KO 1st day *p* = 0.089, 3rd day *p* = 0.4904, 4th day *p* = 0.7607). Two-way ANOVA, *p* = 0.0063 training day’s effect, *p* < 0.0001 genotype effect, Tukey’s multiple comparison test. Right: graph shows the total number of trials to reach the learning criterion for each group. Two-way ANOVA genotype and trials needed effect: *p* = 0.0164. *n* = 8 WT, 6 APP/PS1, 8 APP/PS1/c-Abl KO, and 6 c-Abl KO animals. Data are presented as mean ± SEM. p* < 0.05.

The task procedure consisted of three sessions: habituation, sample, and test. The habituation session was repeated on two consecutive days. On the first habituation day, we recorded animals’ performance in the Open Field, an empty space in which mice are allowed to freely move for 10 min while being recorded, used to evaluate the general activity levels, such as exploration habits and locomotor activity (Seibenhener and Wooten, 2015). The identical to-be-familiarized objects were placed in the apparatus during the sample session. The animal was positioned at the wall’s midpoint opposite the sample objects. After the sample-object exposure time (10 min), the animal was removed and returned to its home cage. The test session was performed 24 h after the sample day. One familiar object and a novel object were placed in the apparatus (Figure 1B). The testing session lasted for 5 min. The exploration time of both objects was measured, and the recognition index (RI) was calculated as the time spent exploring the novel object relative to the total time spent exploring both objects. RI above 0.5 means that the animal can differentiate the novel object from de familiar one. On the other hand, RI below 0.5 means that the animal cannot distinguish between objects.

The task procedure of the OLM is like the NOR procedure. The difference is that one object is displaced to a new position in the test session (Figure 1A). In the OLM, the RI is interpreted as the animal’s capacity to recognize the displaced object. Another difference with the NOR is that the OLM task has intra-maze visual cues. The maze used to perform Open Field, NOR, and OLM tests was a gray acrylic rectangular box (46°×°27 cm). Objects used in the NOR and OLM tests were previously equilibrated (to avoid innate preference) and were distinct in each test.

#### 2.3.2. Barnes maze

In this test, animals should learn and remember the location of an escape hole in an anxiogenic, elevated, and illuminated circular open field (Barnes, 1979; Toledo and Inestrosa, 2010). The maze is a white circular platform of 70 cm diameter elevated at 70 cm from the floor with 20 equally spaced holes along the perimeter (7 cm diameter each) located at 2 cm from the platform’s edge. The platform has 20 equally spaced holes along the perimeter. Visual cues were located on the walls of the room. A black plexiglass escape box (17 × 13 × 7 cm) was located under one of the holes. The maze was illuminated with two incandescent lights to yield a light level of ∼600 lux impinging on the circular platform. The location of the escape box was consistent for a given mouse but randomized across mice (Figure 1C). Each mouse was given four daily trials with an intertrial interval (ITI) of 15 min for four consecutive days. The mouse was placed in the start box for every training trial for 10 s, with the room lights turned off. After time had elapsed, the chamber was lifted, the lights turned on, and the mouse was free to explore the maze. The session ended when the mouse found the hole and entered the escape box or after 3 min. When the mouse entered the escape box, the lights were turned off, and the mouse remained in the dark for 1 min before the next trial began. If the mouse did not find the escape box within 3 min, the experimenter guided the mouse to the escape. We measured latency to enter the escape box (Negrón-Oyarzo et al., 2015).

#### 2.3.3. Morris water maze, memory flexibility, and open field tests

MWM (Morris, 1984) consisted of a round pool made of white plastic of 100 cm diameter, filled to 16 cm depth with water maintained at 19–21°C and colored with white dye to hide the platform. The platform was transparent acrylic (9 cm diameter) in the corresponding quadrant center. Briefly, mice were trained to find the platform three times at different quadrants for 8 days. Animals that found the platform within 60 s were allowed to remain on the platform for 30 s. Those that did not were manually placed on the platform for 30 s.

The MF was performed as described by Toledo and Inestrosa (2010). Each animal was trained in a circular water maze for 4 days. Each day, the platform was changed to the next subsequent quadrant. The animal was considered to finish the training when reaching the criterion: three consecutive trials with an escape latency of 20 s on each training day, up to 15 trials. Once finished testing, the animal was removed from the maze, dried, and returned to its cage.

#### 2.3.4. Data collection

The BM, OLM, and NOR behavioral tests were carried out in the Behavioral Room at the Animal Care facility of the Centro de Investigaciones Médicas (CIM) Each mouse was recorded with a video camera (ImageLab, model CB3200) fixed above the behavioral apparatus. Videos were acquired by Lab View software and analyzed offline using the idTracker video-tracking software (Pérez-Escudero et al., 2014) and Matlab for the analyses. The MWM and MF tests were performed in the Behavioral Room at the Departamento de Biología Celular y Molecular, PUC. The videos were acquired and analyzed using the ANY-maze video tracking system.

### 2.4. Immunofluorescence staining

Mice were anesthetized with a mix of xylazine, ketamine, and acepromazine (4:4:1), then perfused with a peristaltic bomb (Velp Scientifica SP311) with 100 mL of ice-cold PBS and later with 50 mL of PFA 4%. Brains were removed and post-fixed with PFA 4% at 4°C O/N, followed by Sucrose 30% in PBS at 4°C O/N. Brains were cut into a cryostat (Leica CM1850) with 25 μm coronal sections.

Immunofluorescence was done on floating sections. Sections from different brain areas were selected, mainly the hippocampus and prefrontal cortex, and washed with PBS 1X for 10 min. Sections were permeabilized with Triton X-100 0.4% for 30 min. Then, incubated with Glycine 0.15 M for 15 min and fresh NaBH_4_ 10 mg/mL for 15 min, washed with PBS 1X for 10 min, and incubated with NH_4_Cl 50 mM for 10 min. Sections were incubated with blocking solution (Triton X-100 0.4% + BSA 3%) for 1 h, and O/N with primary antibody anti-WO2 (Millipore, MABN10), NeuN (ab177487, Abcam), Sox2 (Cell Signaling, 3728S), Doublecortin (Santa Cruz Biotechnology, sc-271,390) or GFAP (GA5, Cell Signaling, 3670S) (all 1:1000 dilution) at 4°C. The next day, sections were washed 3×10 min, and incubated with anti-mouse 488, the AlexaFluor-conjugated secondary antibody (1:1,000 dilution) for 2 h RT, and Hoechst staining for nuclei visualization. Once finished, the sections were washed 3°×°15 min per time and mounted on a slide.

Fluorescence images were captured under a Zeiss Axioscope 5 microscope. Images were always acquired using the same settings and quantified using ImageJ (Fiji).

### 2.5. Statistics

Data sets were tested for normality using the Kolmogorov–Smirnov test and then compared with the appropriate test. All statistical tests were performed using GraphPad Prism 8 software (GraphPad Software, San Diego, CA, United States). When corresponding, the behavior, plaque’s area, and number were analyzed using one-way ANOVA, two-way ANOVA, three-way ANOVA, Tukey’s *post-hoc* test, and one-sample *t*-test, as indicated in each figure. All statistical assessments were considered significant when *p* < 0.05. All data are expressed as mean ± SEM.

## 3. Results

We have shown that the absence of c-Abl (c-Abl KO) in the brain improves the performance of mice in learning tasks involving spatial memory (González-Martin et al., 2021). To assess the effect of brain-specific genetic ablation of c-Abl on the cognitive performance of 10-month-old AD mice, we carried on a battery of behavioral tests mainly depending on hippocampal activity. Tests were performed sequentially, first Open Field to control locomotor activity and anxiety, followed by Object Location Memory (OLM) and Novel Object Recognition (NOR) which are less stressful tests, and then the Barnes Maze (BM).

We used the OLM task to evaluate spatial memory (Murai et al., 2007). Here, we used the factor Recognition Index (RI) to evaluate the mice’s capacity to recognize the displaced object. As expected, 10-month-old APP/PS1 mice cannot recognize the displaced object (RI: 0.49). Unexpectedly, aged WT mice neither recognized the displaced object (RI: 0.49). Indeed, 4-month-old WT mice recognized the displaced object (Supplementary Figure S1A, RI: 0.60). Thus, this cognitive impairment was associated with mice aging. Interestingly, both aged c-Abl KO and APP/PS1/c-Abl KO mice were able to identify the displaced object (RI: 0.59 and 0.64, respectively). Moreover, there is a significant difference between APP/PS1/c-Abl KO and APP/PS1 groups (Figure 1A).

Literature suggests that adult neurogenesis is reduced in AD in both humans and mice (Babcock et al., 2021), and that the generation of new adult neurons in the dentate gyrus (DG) of the hippocampus specifically, in the subgranular zone (SGZ), affects rat’s performance in behavioral tasks like the NOR (Jessberger et al., 2009). Indeed, it has been described that adult neurogenesis is important for learning and memory (Cancino et al., 2013; Toda et al., 2019). During aging, recognition memory is affected as young, middle-aged and senile rats have compromised object location and recognition-associated memory with marked decrease of adult-born immature neurons in the DG (Canatelli-Mallat et al., 2022). Thus, we addressed adult neurogenesis in brain slices by immunofluorescence for the neural stem cell marker Sox2, and the newly-born neuronal marker DCX (doublecortin) (Supplementary Figure S2), and found no positive cells in aged groups (10 and 22-month-old mice). We also used 3-month-old WT mice as a positive control for neurogenesis, and, as expected, found Sox2+ or DCX+ cells in the SGZ, that were consistently lost in AD mice; further suggesting that the behavioral improvement observed during conditional ablation or pharmacological inhibition of c-Abl is independent of adult neurogenesis.

Therefore, the absence of c-Abl improves a subset of spatial memory: remembering objects and their position in space. Even healthy mice conditionally knock-out for c-Abl in the brain had improved performance (González-Martin et al., 2021). These data indicate that c-Abl absence improves spatial learning and memory performance in AD mice.

To dissect if c-Abl affects hippocampal-associated learning, we also carried out the NOR test, a hippocampus-independent test that evaluates rodents’ recognition memory where the factor RI means the recognition of a novel object (Antunes and Biala, 2012). We found that WT mice could recognize the novel object (RI: 0.62). However, APP/PS1, c-Abl KO (RI: 0.53), and APP/ PS1/c-Abl KO (RI: 0.55) mice did not recognize novelty (Figure 1B). According to previous reports, WT vs. APP/PS1 (RI: 0.44) groups were significantly different, indicating that AD mice had a deficit in the NOR tests (*p* = 0.0315) (Zhang et al., 2012). Our data indicate that c-Abl absence does not improve recognition memory in APP/c-Abl KO-aged animals.

Interestingly, when we evaluated mice behavior in the BM we found that c-Abl absence in APP/PS1 mice reduced the memory decline (Sunyer et al., 2007). The BM is a hippocampus-dependent task assessing rodents’ spatial learning and memory; in which the latency to the escape hole was measured for 4 days. A significant difference between the first and the next 3 days is a learning indicator. Here, we found that WT mice effectively learned the location of the escape hole, showing a significant reduction in latency between the first and the fourth day (149.6 and 78.2 s, respectively, p** = 0.0047). In contrast, APP/PS1 mice were unable to learn (1st day: 134.1 s, 4th day: 89.3 s, *p* = 0.1110) (Figure 1C, Supplementary Figure S1B). Interestingly, c-Abl KO mice showed a marked reduction in latency since day two (1st day: 147.2 s, 2nd day: 75.5 s, p*** = 0.0006, 4th day: 50.9 s, p*** < 0.0001) (Figure 1C). APP/PS1/c-Abl KO mice also showed a significantly reduced latency between the first day and day four (1st day: 124.4 s, 4th day: 56.8 s, p** = 0.0044). The APP/PS1/c-Abl KO and c-Abl KO group presented a better learning performance and showed lower latency than the WT group in the whole test regardless of the acquisition day (Figure 1C).

Finally, the mice’s general motor activity was assessed as control using an Open Field test (Seibenhener and Wooten, 2015), where the animal explored an empty arena. We did not find any differences between groups in locomotor activity measured as traveled distance (*p* = 0.7625) or the time spent in the center zone as an indicator for anxiety-like behavior (*p* = 0.7690), discarding any motor disability or basal stress (Figure 1D). Thus, the differences shown here are just the product of cognitive processes.

To confirm that c-Abl genetic ablation reduces the hippocampal-dependent memory deficits in AD mice we used a new set of mice and applied the Memory Flexibility (MF) test, which is a variation of the Morris Water Maze (MWM) as it has been seen to be more sensitive to hippocampal dysfunction in older mice (Chen et al., 2000). We measured the number of trials to reach the learning criterion over 4 days. As expected, APP/PS1/c-Abl KO mice performed better than APP/PS1 mice, which took more trials to learn the platform’s position on a single experimental day (Figure 1E, left). We found that WT mice needed fewer trials (5.38 ± 0.63 trials from 15 daily trials), while APP/PS1 mice needed almost all attempts (9.71 ± 0.66 trials per day) to reach the criterion. Similar to the WT group, c-Abl KO mice needed a few trials to reach the criterion (5.42 ± 0.86 trials per day). More importantly, the APP/PS1/c-Abl KO mice needed almost half of the total trials to reach the criterion (6.91 ± 0.52 trials per day) and were no different from WT (Figure 1E, right). With this more sensitive test, we observed a clear effect of c-Abl absence in AD preventing cognitive decline.

Altogether, our results suggest that the effect of c-Abl absence on cognition is selective, mostly impacting memories that depend on the hippocampus.

### 3.1. A neurotinib diet improves hippocampal-dependent memory in AD, the same as c-Abl absence in AD

To continue our investigation, we assessed spatial learning and memory in AD mice, both conditional knock-out for c-Abl in the brain or fed with neurotinib, a recently designed and disclosed allosteric c-Abl inhibitor with improved CNS permeability (Marin et al., 2022), to perform additional hippocampus-dependent tests.

In mice, neurotinib has preferential distribution toward CNS reaching single-digit micromolar levels in the brain and a t½ in the range of 8 h upon oral gavage administration at 12.5 mg/kg. Similar single-digit micromolar levels are constantly maintained in the brain after supplementing the molecule in the chaw diet at 67 ppm for 2 weeks, equivalent to daily ingestion of 12.5 mg/kg of neurotinib (Marin et al., 2022, Supplementary Figure S5). Therefore, to approximate our study to the AD clinical testing and pharmaceutical approach, we evaluated “elder” mice as considered by JAX laboratory around 18–24 months old, similar to 56–69 years human (Flurkey et al., 2007), after feeding them for a total of 6 months with a neurotinib-containing diet.

**Figure 2:**
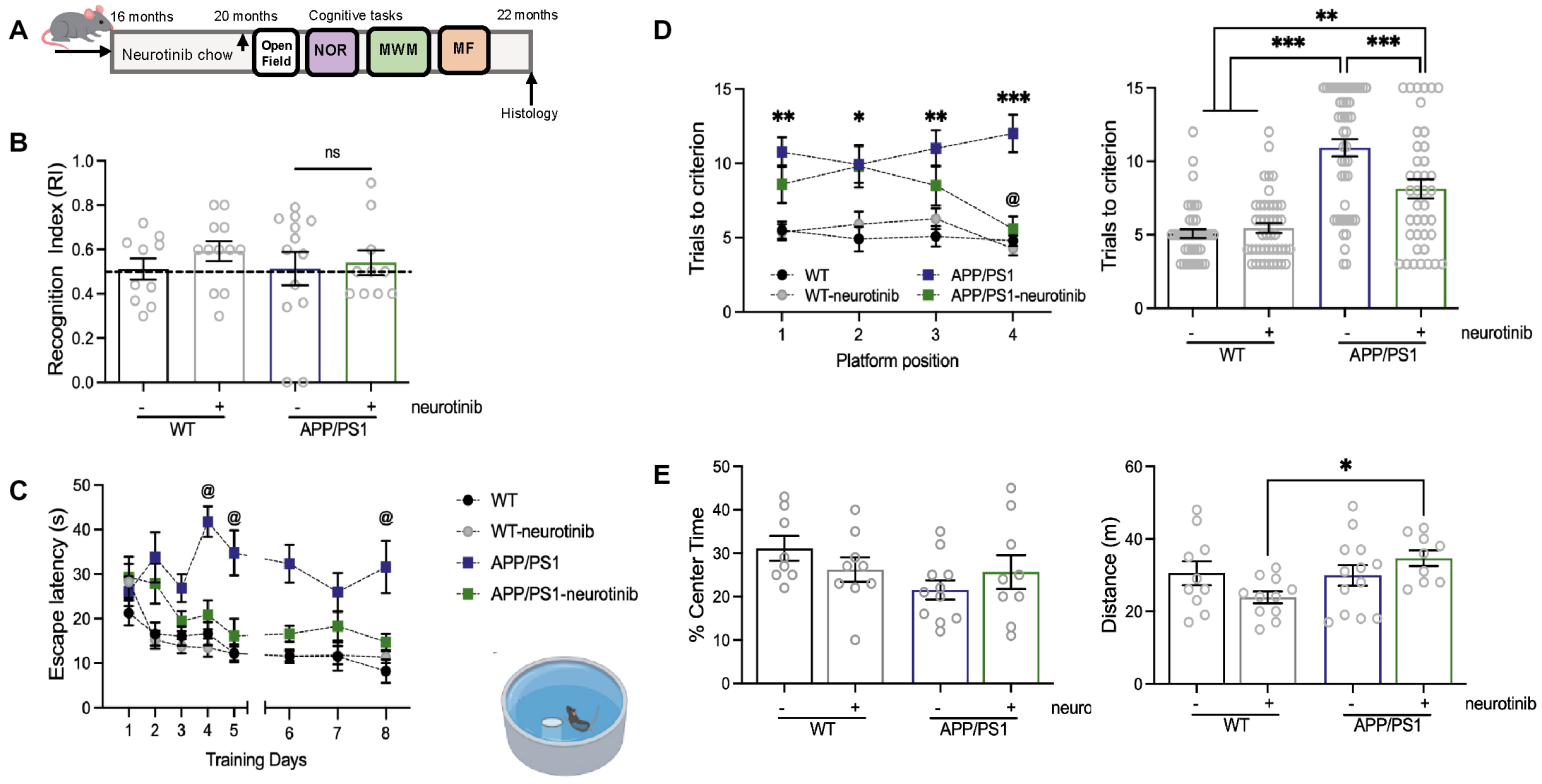
c-Abl absence and neurotinib-diet improve the cognitive performance of AD mice in hippocampus-dependent tasks. **(A)** WT and APP/PS1 mice were fed for 4 months with a control diet or diet containing 67 ppm of neurotinib before starting the cognitive tests. At 20-month-old, mice were subjected to learning tasks with resting periods between them, and maintenance of *ad libitum* respective diets until tissues were collected. **(B)** Novel Object Recognition test. Graph shows the time spent around the new object, compared to the total exploration time. The dotted line represents the chance. Two-way ANOVA genotype effect: *p* = 0.9573, treatment effect: *p* = 0.5689. **(C)** Morris Water Maze test. Graph shows the time to find the platform during 8-training days (escape latency) @ mean significantly different from 0.5. α: days in which APP/PS1 is different in comparison to another group. Three-way ANOVA, Tukey *post-hoc* multiple comparison test, genotype effect: *p* < 0.0001, treatment effect: *p* = 0.0361, day: *p* < 0.0001, days × treatment: *p* = 0.0237. **(D)** Graphs show the memory flexibility task results as the number of attempts to find the platform in each position at different training days (left), and the number of trials to reach the learning criterion for each group (right, treatment and acquisition day effect *p* = 0.1195, neurotinib effect *p* < 0.0001). Significance was annotated for APP/PS1 vs. WT mice (1st day p** = 0.0016, 2nd day p** = 0.016, 3rd day p** = 0.0033, 4th day p** = 0.0033). @ mean significant different between APP/PS1 vs. APP/PS1-neurotinib fed mice p** = 0.0027. Two-way ANOVA, Tukey’s *post-hoc* multiple comparison test. **(E)** Open field test for anxiety-like behavior (left, *p* = 0.8962) and locomotor activity evaluation (right, *p* = 0.7167) of neurotinib-fed mice. Bar shows the percent of time spent exploring the center zone, and the distance traveled during the whole exploratory time. Two-way ANOVA, Tukey *post-hoc* multiple comparison test: WT-neurotinib vs. APP/PS1-neurotinib: p* = 0.0378. Same *n* = 10 WT-control diet, 12 APP-control diet, 11 WT-neurotinib diet, and 10 APP-neurotinib fed animals for each test. Data are shown as mean ± SEM.

As before, we sequentially performed the learning tasks: Open Field to control locomotor activity and anxiety, followed by NOR, less stressful tests. Then the MWM, a task broadly used to evaluate spatial memory, and finally the more sensitive MF test (Figure 2A). As for the genetic ablation of c-Abl in AD mice, we found no significant difference among groups in animals fed with neurotinib when we evaluated recognition memory in the NOR test (Figure 2B). Like APP/PS1/c-Abl KO animals (Figure 1B), we found no changes in the time spent recognizing novelty in neurotinib-fed WT or APP/PS1-aged fed mice (RI: 0.59 and 0.54, respectively, *p* = 0.9129). Therefore, neither c-Abl ablation nor neurotinib improves mice’s performance in hippocampus-independent tasks.

We then performed the MWM, a task broadly used to evaluate spatial memory (Morris, 1984; Vorhees and Williams, 2006), in which amyloid pathology causes impaired performance in 12-month-old mice (Lalonde et al., 2005), and found that WT mice had significantly reduced escape latency across training days than APP/PS1 (5th day 12.21 ± 2.0 s vs. 34.77 ± 5.0 s, p* = 0.0104; 8th day 8.23 ± 2.6 s vs. 31.61 ± 5.9 s, respectively, p* = 0.0217) (Figure 2C). APP/PS1 mice displayed poor performance, while neurotinib-fed APP/PS1 mice significantly reduced escape latency across training days (5th day 16.15 ± 3.8 s, p* = 0.0477; 8th day 14.78 ± 1.8 s, *p* = 0.0958). No differences were found among WT, WT-neurotinib, and APP/PS1-neurotinb groups since day 3 of the MWM test. Interestingly, WT-neurotinib mice had no differences from WT mice (5th day 12.20 ± 1.6 s, *p* > 0.9999; 8th day 11.34 ± 1.3 s, *p* = 0.7241), meaning that the neurotinib diet did not modify basal performance in WT mice (Figure 2C).

Importantly, the MF analysis for APP/PS1-neurotinib-fed mice showed the same trend as the MF for APP/PS1/c-Abl KO (Figure 1E). Interestingly, in the MF mice had to find the hidden platform in a different location each day. Thus, at day 4, APP/PS1-neurotinib fed mice showed short-term memory improvements (1st vs. 4th-day trial p* = 0.0353), and behaved significant different from APP/PS1 mice (p** = 0.0027) (Figure 1D, left). We also found that WT-neurotinib-fed mice and WT mice needed fewer trials to reach the criterion (5.5 ± 0.3 and 5.1 ± 0.3 trials, respectively), while APP/PS1 mice needed 2-times more trials (10.9 ± 0.6 trials) to reach the criterion (Figure 2D, right). More interestingly, APP/PS1-neurotinib-fed mice needed significantly fewer trials to reach the hidden platform than APP/PS1 mice to reach the criterion (8.1 ± 0.6 trials) (Figure 2D, right). As in BM and OLM tests, the MF data suggest that c-Abl absence or its inhibition improves hippocampus-dependent memory.

As before, we used the Open Field test for mice’s general motor activity assessment and found no differences in their anxiety-like behavior (*p* = 0.1385, Figure 2E, left). Overall, mice’s locomotor activity was not affected by the neurotinib diet (*p* = 0.7167). The APP/PS1 model showed hyperactivity as animals were prone to walk around and away from the center; a characteristic described for APP animals with the APP Swedish and PSEN1 mutation (Lalonde et al., 2005). Similarly, APP/PS1-neurotinib fed mice traveled longer distances (34.67 ± 2.2 m) (Figure 2E, right). However, WT-neurotinib fed mice traveled a few meters of the empty arena in the same amount of time (23.82 ± 1.6 m, p* = 0.0378), in correlation with their reduced walking speed (Supplementary Figure S3). Further suggesting increased hyperactivity for the APP/PS1-neurotinib fed mice instead of motor affections with the chow.

Altogether, the data presented here indicate that treating an animal model of AD with high brain penetrant c-Abl inhibitor neurotinib improves memory performance in a hippocampus-dependent task, replicating the same pattern observed in the AD mice model knock-out for c-Abl.

### 3.2. APP/PS1/Abl-KO mice and neurotinib-fed mice have reduced accumulation of Aβ plaques in the brain

**Figure 3:**
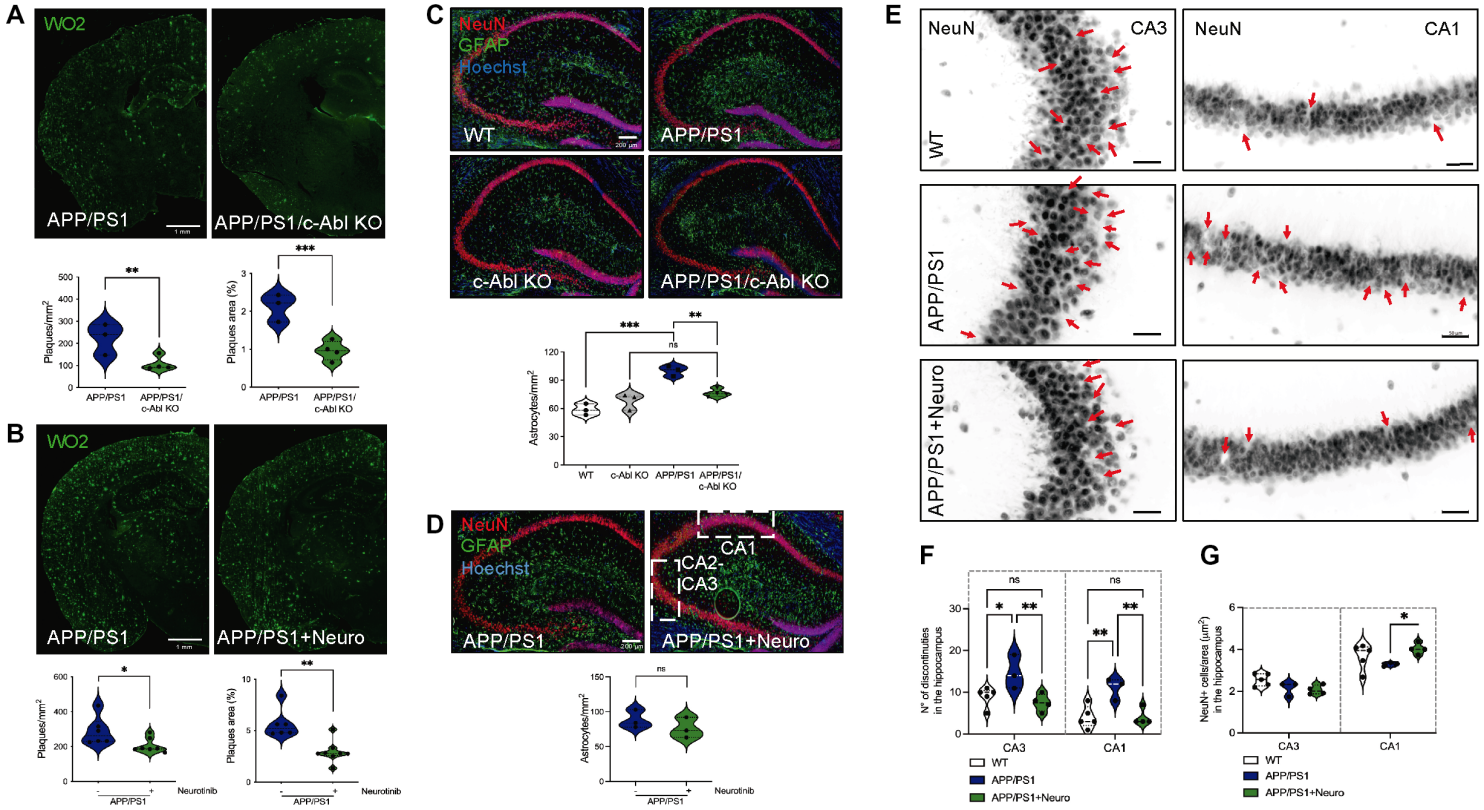
c-Abl absence and inhibition reduced amyloid-beta burden, neuroinflammation, and reduced neuronal loss in the APP/PS1 mice model of AD. **(A,B)** Representative immunofluorescences from coronal brain sections stained with WO2 antibody for amyloid-beta plaques in green. Graph shows the number of amyloid-beta plaques per mm^2^ and the percentage area of each plaque in relation to the cortex and hippocampus brain area. *n* = 4 animals, 2 slices per genotype or treatment. Scale bar = 1 mm. **(A)** 10-month-old APP/PS1 and APP/PS1/c-Abl KO mice, *n* = 3–4 animals per genotype. Two-way ANOVA, *post-hoc* Tukey’s multiple comparison test, p** = 0.0073 for plaques/mm^2^, left; p*** = 0.0005 for plaques area (%), right. **(B)** 22-month-old APP/PS1 mice fed with control or neurotinib diet. Non-parametric Mann–Whitney *t*-test p* = 0.0350 for plaques/mm^2^, left; p** = 0.0040 for plaques area (%), right. **(C,D)** Representative immunofluorescences from coronal brain sections for the astrocyte marker GFAP (green), NeuN marker for neurons in the hippocampus (red), and the nucleus (Hoechst, blue). Graphs show the number of astrocytes per mm^2^. *n* = 3–4 animals per genotype or treatment. Scale bar = 200 μm. **(C)** 10-month-old WT, c-Abl KO, APP/PS1, and APP/PS1/c-Abl KO mice. Two-way ANOVA, *post-hoc* Tukey’s multiple comparison test, c-Abl absence effect *p* = 0.0651. c-Abl KO vs. APP/PS1/c-Abl KO *p* = 0.3509. **(D)** 22-month-old APP/PS1 mice fed with control or neurotinib diet. Non-parametric Mann–Whitney *t*-test *p* = 0.4. **(E)** Same immunofluorescences for the neuronal marker in **(D)**, NeuN, of 22-month-old mice fed with control or neurotinib diet are presented in grayscale. Red arrows indicate the discontinuities found in the CA3 and CA1 areas of the hippocampus augmented from the coronal brain sections shown in **(D)** (dotted white square). *n* = 3–5 animals per condition. Scale bar = 10 μm. **(F)** Graph shows the number of discontinuities in the CA3 or CA1 hippocampi. Two-way ANOVA, *post-hoc* Tukey’s multiple comparison test, p*** = 0.0001 treatment effect. **(G)** Graph shows the total number of NeuN^+^ positive cells counted and normalized per CA3 or CA1 areas (μm^2^). Two-way ANOVA, *post-hoc* Tukey’s multiple comparison test, *p* = 0.1335 treatment effect. Data are presented as mean ± SEM. p*** < 0.0001.

It has been shown that c-Abl overexpression in the mouse brain is responsible for gliosis and neuronal loss (Schlatterer et al., 2011), which can be further detrimental in AD. Thus, we analyzed the pathological hallmarks of AD: Aβ aggregation, astrogliosis, and neuronal loss in relation to the genetic ablation of c-Abl and neurotinib-diet. As expected, 10-month-old APP/PS1 mice brain sections involving the brain cortex and hippocampus showed widespread Aβ accumulation as WO2-positive amyloid plaques (Figure 3A, *p* < 0.0001) compared with WT and c-Abl KO control animals. Also, APP/PS1 mice had important astrogliosis followed by GFAP-positive cells (Figure 3C, p*** < 0.0001). Interestingly, APP/PS1/c-Abl KO mice display significantly less number of WO2-positive amyloid plaques, with reduced area in the brain (APP/PS1: 224 ± 40.6 mm^2^ vs. APP/PS1/c-Abl KO: 107.5 ± 16.2 mm^2^, p** = 0.0048) (Figure 3A, left). Also, less astrocytosis, at similar basal levels of c-Abl KO and WT mice (c-Abl KO: 68 ± 5.0 astrocytes/ mm^2^; APP/PS1: 100 ± 3.2 vs. APP/PS1/c-Abl KO: 76.25 ± 2.1 astrocytes/mm^2^, p** = 0.0031) (Figure 3C). Thus, data suggest that the absence of c-Abl could reduce the Aβ burden in the AD mice model.

Studies in APP/PS1 mice and AD patients treated with c-Abl inhibitors like imatinib and nilotinib diminished Aβ deposits in the brain and blood (Cancino et al., 2008; Estrada et al., 2016; Turner et al., 2020). As before, we analyzed similar brain sections of APP/PS1 control and neurotinib-fed mice and found a significant reduction in the amount and area of WO2-positive amyloid plaques (APP/PS1: 288 ± 33 mm^2^ vs. APP/PS1 + neurotinib: 205.7 ± 15.9 mm^2^, p* = 0.0350) (Figure 3B). Although no differences were found in the amount of GFAP-positive cells (Figure 3D, *p* = 0.4), APP/PS1 seem to have more GFAP-positive cells when compared with WT controls, but again, non-significant in the reduced sample analyzed (Supplementary Figure S4, *p* = 0.0571).

Finally, we analyzed the neuronal loss using the NeuN marker for mature neurons in APP/PS1 mice fed with the control or neurotinib-supplemented chow (Figure 3E). No differences were found in the dentate gyrus of the hippocampus (Supplementary Figure S5). As expected, the hippocampi of APP/PS1 animals showed an increased number of discontinuities in CA1–CA3 areas (CA1: WT: 4 ± 1.2 vs. APP/PS1: 11 ± 1.5, p** = 0.0046) (Figures 3E,F). Interestingly, APP/PS1-neurotinib fed mice had reduced neuronal loss, similar to WT levels (CA1: 4 ± 1, p** = 0.0065). In accordance, the number of NeuN^+^ cells per CA1 area was maintained (CA1: APP/PS1: 3.3 ± 0.1 vs. APP/PS1 + neurotinib: 4.03 ± 0.3 cells/μm^2^, p* = 0.0442) (Figures 3E–G). The number of NeuN^+^ cells was not altered in the hippocampi CA3 area (*p* = 0.9621).

Altogether, our results have shown a positive effect of the allosteric inhibitor for c-Abl, neurotinib, over the AD pathology displayed by aged 22-month-old APP/PS1 mice, further validating c-Abl as a key player in the development of AD pathology and cognitive impairment of AD.

## 4. Discussion

Herein we have found that the genetic ablation and inhibition of c-Abl improves the cognitive performance in hippocampus-dependent tasks of AD and healthy mice. The hippocampus plays a crucial role in learning, memory, and spatial navigation and is the first structure to show senile plaques and tau tangles, parameters correlated with AD patients’ cognitive decline (Lane et al., 2018). Thus, our work encourages research of c-Abl as a feasible candidate for memory improvement in AD.

Previous reports have shown that deficiency of some proteins activated in the AD brain that are linked with the progression of AD pathology can reduce the memory alterations characteristic of transgenic mouse models of AD. Active caspase-6 is associated with AD pathological lesions and is present at the early stages of tangle formation. Behavioral examinations of the 5xFAD mouse AD model with caspase-6-KO showed improved spatial learning, memory, and anxiety/risk assessment behavior (Angel et al., 2020). The p75 neurotrophin receptor (p75NTR) has been increasingly co-localized with hyperphosphorylated tau in AD brains, and one report shows that p75NTR-KO diminished the MWM latency in AD mice (Mañucat-Tan et al., 2019). The PLD1 ablation and chronic inhibition using a well-tolerated PLD1-specific small molecule inhibitor prevent the progression of synaptic dysfunction during early stages in the 3xTg-AD mouse model (Bourne et al., 2019).

As widely reported in the literature, the APP/PS1 model displayed a notorious deficiency in recognition memory and different components of spatial memory (Figure 1, Sierksma et al., 2014; Cheng et al., 2019; Li et al., 2019). Nevertheless, the new model, APP/PS1/c-Abl KO mice, has significantly improved performance in all spatial memory tests, indicating that c-Abl absence attenuates AD-associated memory impairment.

When c-Abl was inhibited with neurotinib in a daily diet, none of our groups showed differences in the NOR test nor APP/PS1/c-Abl KO mice. All groups displayed similar values near the chance. WTs spent 11 s on average in the familiar object and 13 s in the novel object. APP/PS1 spent 16 s on average in the familiar object and 23 s in the novel object. Further suggesting that their natural curiosity behavior is unexpected at 22 months old. As seen by Yang et al. (2019), aging severely impacts working memory recognition in 19–21 months old mice. This result was not unpredictable since the NOR test evaluates recognition and working memory and the central brain region involved in the perirhinal cortex (Antunes and Biala, 2012). Microinjections of anisomycin, a protein synthesis inhibitor, in the perirhinal cortex impaired the NOR test. However, the same injection on the hippocampus does not alter recognition memory (Balderas et al., 2008). On another hand, BM is a well-known hippocampus-dependent task that evaluates spatial learning and memory (Barnes, 1979) as well as the MF test (Chen et al., 2000). Therefore, it is very interesting that in the absence of c-Abl, prominently expressed in the hippocampus and cerebellum (Experiment Detail: Allen Brain Atlas: Mouse Brain, n.d.), APP/PS1 mice overcome the characteristic memory impairment of AD. On the 4th day of the BM, WT vs. APP/PS1 latency was 78.2 vs. 89.3 s; with mice behaving differently among both groups. When we dissected their behavioral response to the BM, we found that the 8 WTs behave evenly in a range of 119.5–36.3 s. Only one did not learn the location of the escape hole (Supplementary Figure S1B, gray). While APP/PS1 mice were divided into two populations: 4 mice from 179.3–114 s, a group that did not learn the location of the escape hole; and one population of 4 mice that indeed learned the location of the escape hole with a latency of 71 to 34 s. However, we have considered one mouse that started well but did not learn (Supplementary Figure S1B, light blue), as behavior followed Gaussian distribution. APP/PS1 mice showed variable responses that suggest adding new animals, as we cannot also discard that 10-month-old APP/PS1 mice could be at different stages of the pathology; effect that might cause the differences observed in APP/PS1 population.

c-Abl-KO 4-month-old young mice displayed improved memory performance in the MWM and BM (González-Martin et al., 2021). Similarly, 10-month-old c-Abl KO mice still performed better than WT mice, even in the APP/PS1 model. Interestingly, we show that old WT mice do not recognize the displaced object in the OLM test, but c-Abl KO does. It has been described that hippocampal function declines age-dependently, and WT-aged mice performed worse than WT-young in the OLM test (Murai et al., 2007; Wimmer et al., 2012). Since the c-Abl KO mice recognized the displaced object, not like the age-matched group, it suggests that c-Abl absence reverts the impairment associated with aging, but its absence or kinase activity did not alter adult neurogenesis as a factor for memory improvement. Further, the impairment presented in aged WT mice in the OLM test could be related to task characteristics. The OLM relies on an animal’s innate preference for novelty without additional external reinforcement (Denninger et al., 2018). BM and MF tests use negative external reinforcement to promote spatial learning. In each case, rodents seek an escape from an aversive situation, exposed on a brightly-lit platform or immersed in water, respectively (Denninger et al., 2018). Thus, the cognitive decline observed in the WT group performing the OLM is a typical feature of aged WT mice, abolished in more challenging tasks.

Studies with c-Abl inhibitors have shown improvement in the cognitive status of animal models of AD (Cancino et al., 2008; Estrada et al., 2016; La Barbera et al., 2021). Our laboratory previously showed that treatment with the c-Abl inhibitor, imatinib, reduces path length and escape latency in the MWM of Aβ fibrils injected in rat’s hippocampi and APP/PS1 mice (Cancino et al., 2008). Also, imatinib prevented LTP impairment induced by Aβ oligomers in hippocampal slices (Vargas et al., 2014). Other authors have also shown amelioration of the AD phenotype with nilotinib, which is perceived to be the most CNS penetrating c-Abl inhibitor among the FDA-approved drugs (Turner et al., 2020; La Barbera et al., 2021). However, while animal evaluation of FDA-approved c-Abl inhibitors can be carried out at high doses, clinically observed on-target and off-target toxicities limit the maximal human-tolerated doses for these drugs. Cerebrospinal fluid data upon 6-month administration of nilotinib at its maximal daily tolerated dose during the clinical evaluation with AD patients indicated that this drug is only able to achieve CNS levels 10–20 times below its IC50 against c-Abl, pointing toward severe limitations for the use of FDA-approved c-Abl inhibitors to treat neurodegenerative disorders (Turner et al., 2020). To address this liability, here we evaluate a novel allosteric c-Abl inhibitor, neurotinib, with high brain penetration at a low dose and found that AD mice fed with this inhibitor had reduced escape latency and required fewer trials to reach the learning criterion, improving the cognitive decline impairments seen in AD mice (Figure 2).

The extracellular accumulation of Aβ plaques is a significant histopathological marker of AD (Atwood and Bowen, 2015) as the increased neuroinflammation and astrogliosis are presented in AD mouse models and patients (Schlatterer et al., 2011). In correlation with memory improvements, we have found that APP/PS1/c-Abl KO mice had significantly fewer amyloid plaques and astrogliosis than APP/PS1 in the brain cortex and hippocampus. The same was observed in neurotinib-fed mice (Figures 3A–D). Since c-Abl activity regulates the APP proteolytic processing (Yáñez et al., 2016), and its inhibition has been correlated with the decrease of amyloid burden in *in vivo* models of AD (Cancino et al., 2008; Estrada et al., 2016), a reduction of Aβ toxic species could underlie the reduction in memory impairments of AD mice. On the other hand, Aβ oligomeric species are the first to produce synaptic dysfunction by damaging dendritic spines and removing synaptic contacts (Gomez-Isla et al., 2008; Viola and Klein, 2015). In this context, we have seen that c-Abl participates in synaptic contact removal, increasing susceptibility to AβOs damage (Vargas et al., 2014; Gutiérrez et al., 2019). The resilience given by c-Abl absence might be present in aging and AD as 10-month-old mice showed differences (Supplementary Figure S1). However, it remains to be addressed. Also, it has been recently reported that the inhibition of ABL family leads to increased synaptic strength, especially since Aβ_1-42_ species reduce spontaneous synaptic activity (Reichenstein et al., 2021). Moreover, the expression of active c-Abl in adult mouse forebrain neurons leads to neuronal loss, neuroinflammation, and interferon signaling pathways activation, especially, in the CA1 region of the hippocampus (Schlatterer et al., 2011, 2012). Interestingly, our results showed that inhibiting c-Abl with neurotinib protects neurons from damage, maintaining levels of NeuN^+^ cells similar to WTs, in correlation with fewer discontinuities in the CA1–CA3 regions of the hippocampus (Figures 3E–G).

These results fully support c-Abl as a feasible therapeutic target for AD and validate neurotinib as a preclinical development candidate.

## Data availability statement

The datasets presented in this study can be found in online repositories. The names of the repository/repositories and accession number(s) can be found at: 10.6084/m9.figshare.22217734.

## Ethics statement

The animal study was reviewed and approved by Bioethics and Care of Laboratory Animals Committee of the Pontificia Universidad Católica de Chile and CIBEM. The animal study protocol #170616008 was approved by the Bioethics and Care of Laboratory Animals Committee of the Pontificia Universidad Católica de Chile and CIBEM.

## Author contributions

RL, PF, and AÁ contributed to the conception and design of the study. RL and CP performed all the learning tests in APP/PS1 x c-Abl animals. CM and NE analyzed data from learning tests. CP performed all the experiments in neurotinib-fed mice. AD synthesized the neurotinib chow. CP and CR performed WO2 and GFAP immunofluorescences. BC and GC performed DCX and Sox2 immunofluorescences. CF, DC, and AG-M generate the APP/PS1/c-Abl KO transgenic mice strain. RL and DG wrote the first version of the manuscript and discussed the results. DG analyzed data and generated the figures. JM and SZ wrote sections of the manuscript.

## Funding

This work was supported by the Agencia Nacional de Investigación y Desarrollo (ANID): Fondecyt grant [1201668 (AÁ)], Fondecyt [grants 1190334 and 1230337 (SZ)], Fondecyt [grant 1210507 (GC)], Fondef [grant ID21I10347 (AÁ, SZ)], Fondef [grant D10E1077 (AÁ, SZ)], Program ICM-ANID-ICN2021 045 (AÁ). Part of this work was supported by intramural funding of the National Center for Advancing Translational Sciences.

## Conflict of interest

The authors declare that the research was conducted in the absence of any commercial or financial relationships that could be construed as a potential conflict of interest.

Neurotinib is under the patent WO2019/173761 A1.

## Publisher’s note

All claims expressed in this article are solely those of the authors and do not necessarily represent those of their affiliated organizations, or those of the publisher, the editors and the reviewers. Any product that may be evaluated in this article, or claim that may be made by its manufacturer, is not guaranteed or endorsed by the publisher.
