# Supplementary material for: c-Abl tyrosine kinase down-regulation as target for memory improvement in Alzheimer’s disease
Source: Front Aging Neurosci. 2023 Jun 5;15:1180987. doi: 10.3389/fnagi.2023.1180987 (PMC10289333; doi:10.3389/fnagi.2023.1180987)
Supplement: Supplementary file 2 [file Data_Sheet_1.docx]

Supplementary Material

c-Abl tyrosine kinase down regulation as target for memory improvement in Alzheimer’s disease

Rilda León^1,†^, Daniela A. Gutiérrez^1†^, Claudio Pinto^1^, Cristian Morales^2^, Catalina de la Fuente^1^, Cristóbal Riquelme^1^, Bastián I. Cortés^3^, Adrián González-Martin^1^, David Chamorro^1^, Gonzalo I. Cancino^3^, Silvana Zanlungo^4^, Andrés E. Dulcey^5^, Juan J. Marugan^5^, Alejandra Álvarez R^1,^*

*** Correspondence:** Corresponding Author: Alejandra Álvarez Rojas, [aalvarez@bio.puc.cl](mailto:aalvarez@bio.puc.cl)

# Supplementary Figures


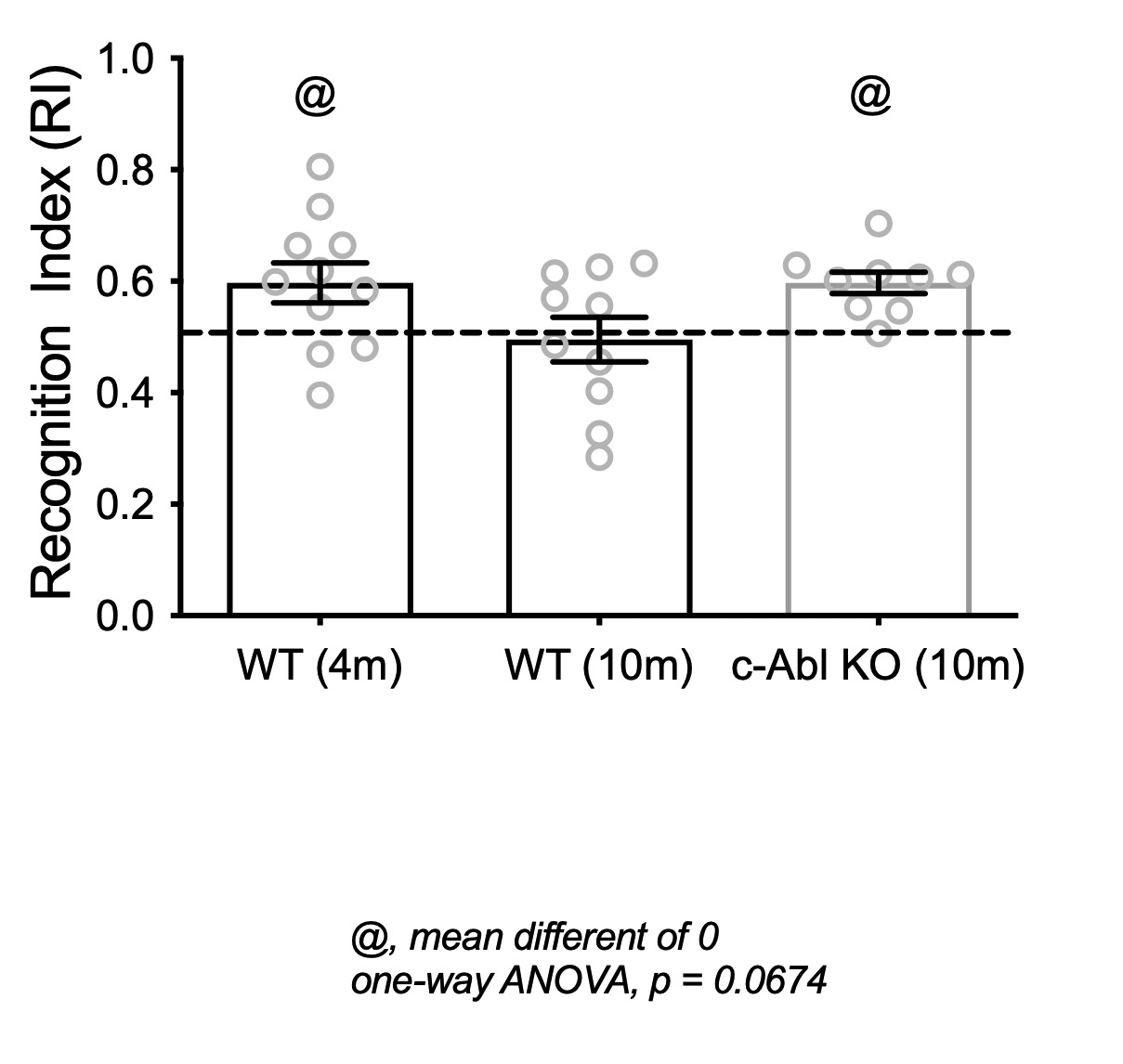

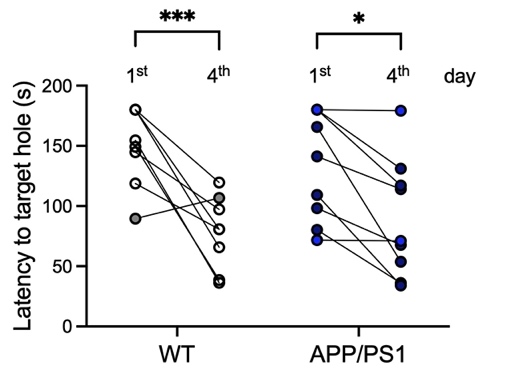


A

B

**Supplementary Figure 1. Age affects wild-type performance in the Object Location Memory and Barnes Maze tests. A.** The bars show the time that animals spent in the relocated object, compared to the total time of exploration (recognition index) in the Object Location Memory (OLM) test. Data is presented for young (4 month-old, 4m) and old (10 months, 10m), wild-type (WT) and c-Abl knock-out (c-Abl KO) animals. The dotted line represents the chance. t-test, p=0.0303; @ mean significant different from 0.5, the chance. **B.** Graph shows the time to reach the escape hole (latency) in the Barnes Maze during the first (1^st^) vs the last day (4^th^) of the test for each WT (149.6 vs 78.2, p***=0.0004) and APP/PS1 (134.1 vs 89.3, p*=0.0116) animal analyzed. Gray and light-blue dots represent animals that did not learn nor changed behavior among days. Data are presented as mean±SEM.

**
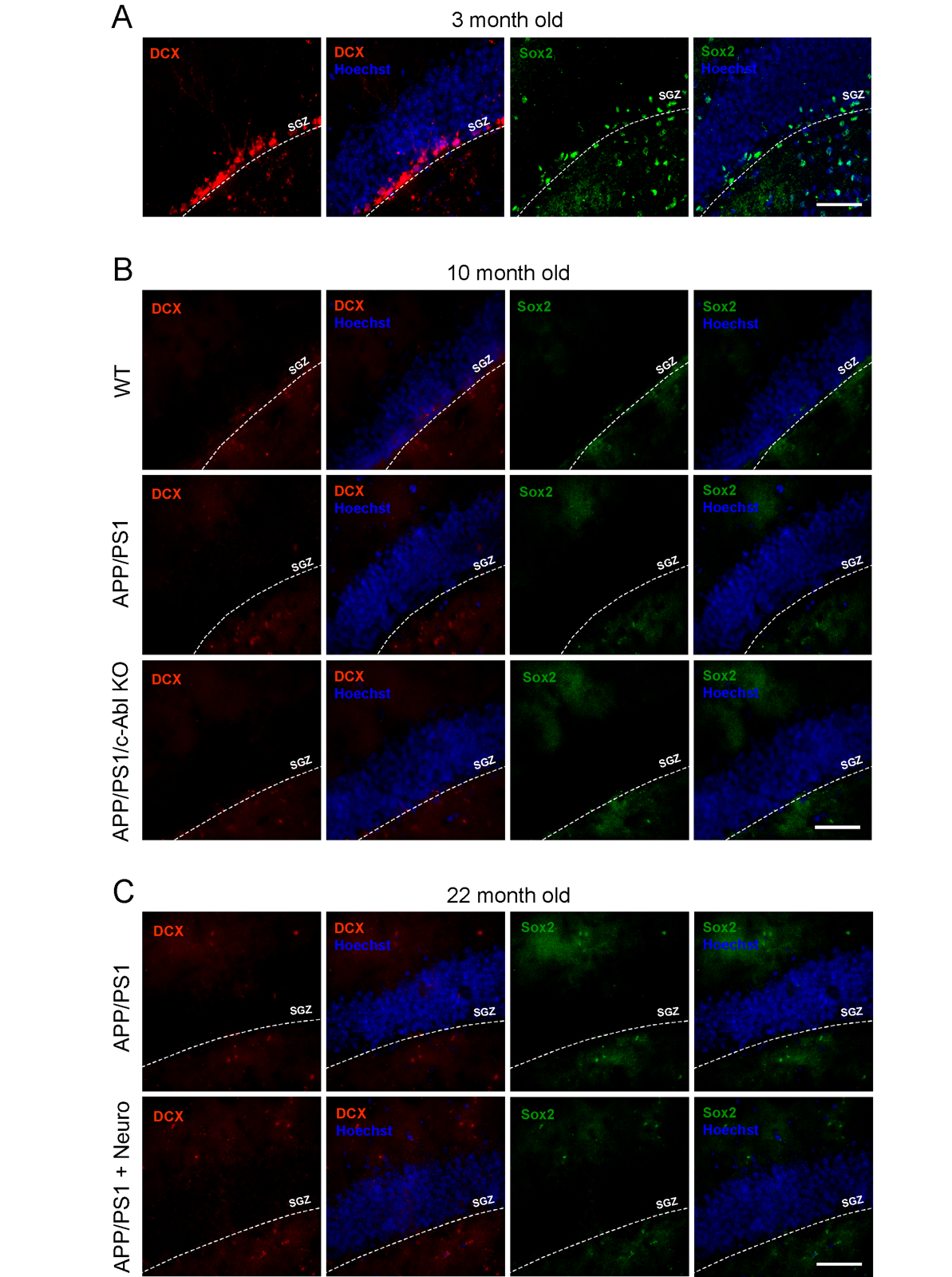
**

**Supplementary Figure 2. Improved learning is independent of adult neurogenesis.** Brain coronal sections were antibody stained for DCX (doublecortin, red) for newly-born neurons, and Sox2 (green) to label the neural stem cell population by immunofluorescence. Hoechst staining (blue) was used to label all cells. The subgranular zone (SGZ) is underlined with a white dotted line. **A.** 3 month-old brain coronal sections were used as positive control. **B, C:** WT and APP/PS1 mice conditionally deficient for c-Abl (c-Abl KO) in the brain at 10 month-old (B), and 22 month-old APP/PS1 mice fed with control or neurotinib-supplemented diet at 60 ppm during the previous four months (C). n= 3 sections per 3 animals per genotype and treatment. Scale bar = 50 µm.


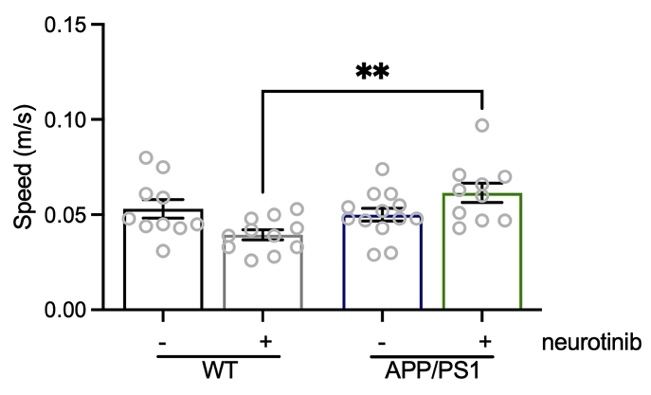


**Supplementary Figure 3. Average walking speed is affected as a result of genotype plus neurotinib diet.** WT and APP/PS1 mice were fed for four months with control diet or diet containing 67 ppm of neurotinib before starting the cognitive tests. At 20-month-old, mice were subjected to learning tasks with resting periods between them, and maintenance of *ad libitum* respective diets until tissues were collected. Mice’s walking speed was measured during the Open Field test. APP/PS1-neurotinib fed mice vs WT-neurotinib fed mice p=0.0025 Two-way ANOVA, Tukey’s post-hoc multiple comparison test. n= 10 WT-control diet, 12 APP-control diet, 11 WT-neurotinib diet, and 10 APP-neurotinib fed animals for each test. Data are shown as mean ± SEM.

**
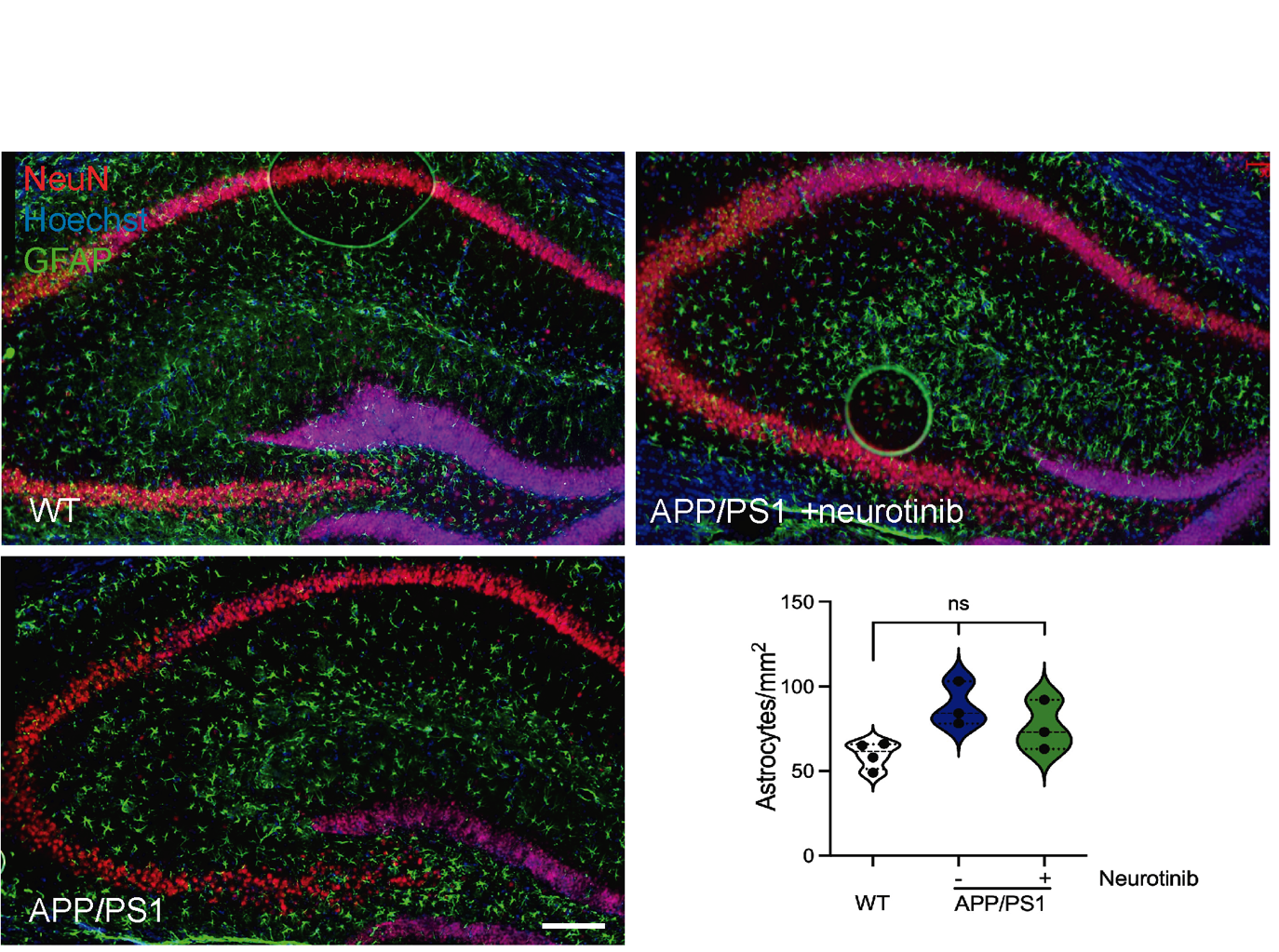
**

**Supplementary Figure 4. Astrocytosis seems to be altered in APP/PS1 mice.** 22-month-old WT and APP/PS1 mice fed with control or neurotinib diet. Representative immunofluorescences from coronal brain sections stained with GFAP (green), NeuN marker for neurons in the hippocampus (red), and the nucleus (Hoechst, blue). Graphs show the number of astrocytes per mm^2^. n=3-4 animals per genotype or treatment. Scale bar = 200 µm.


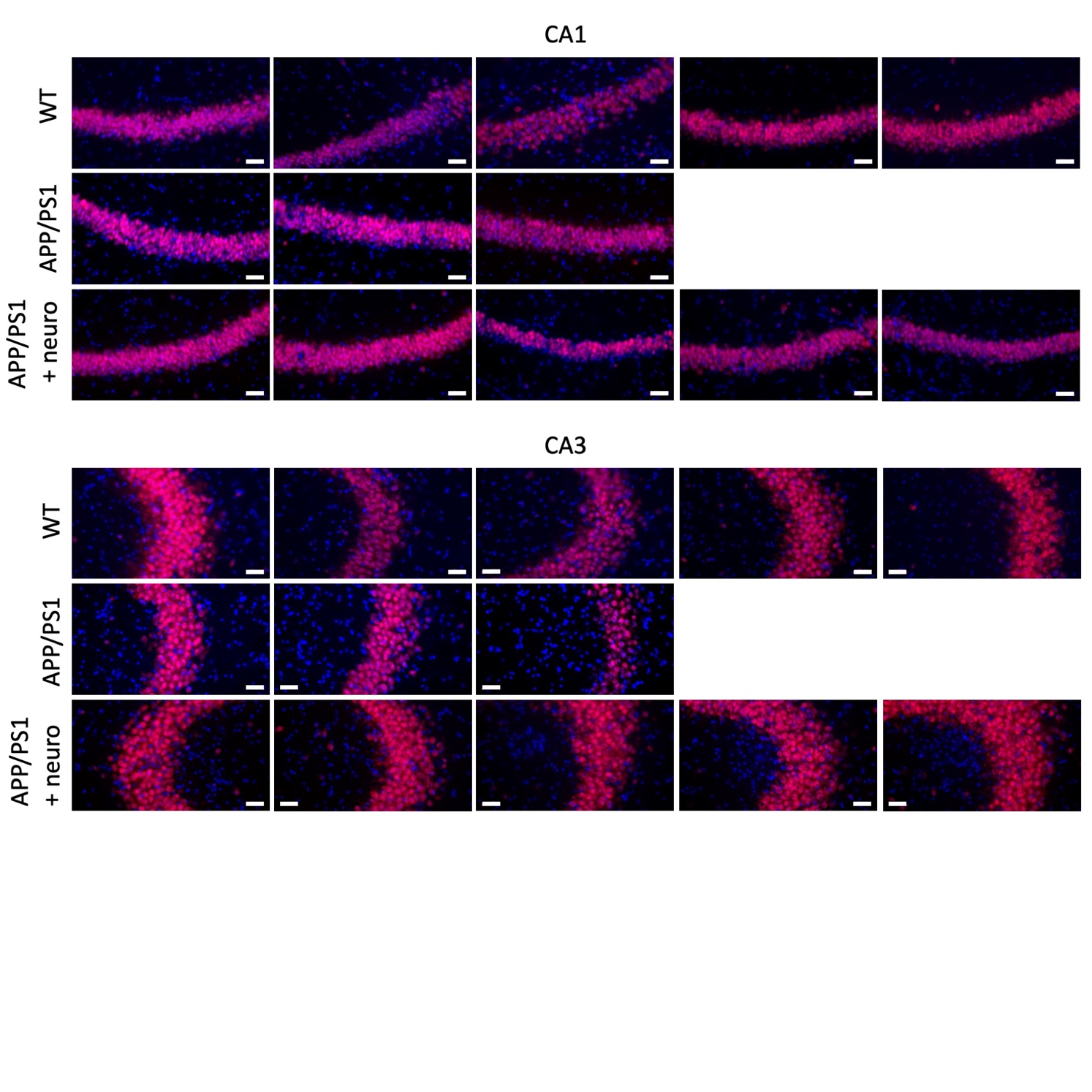

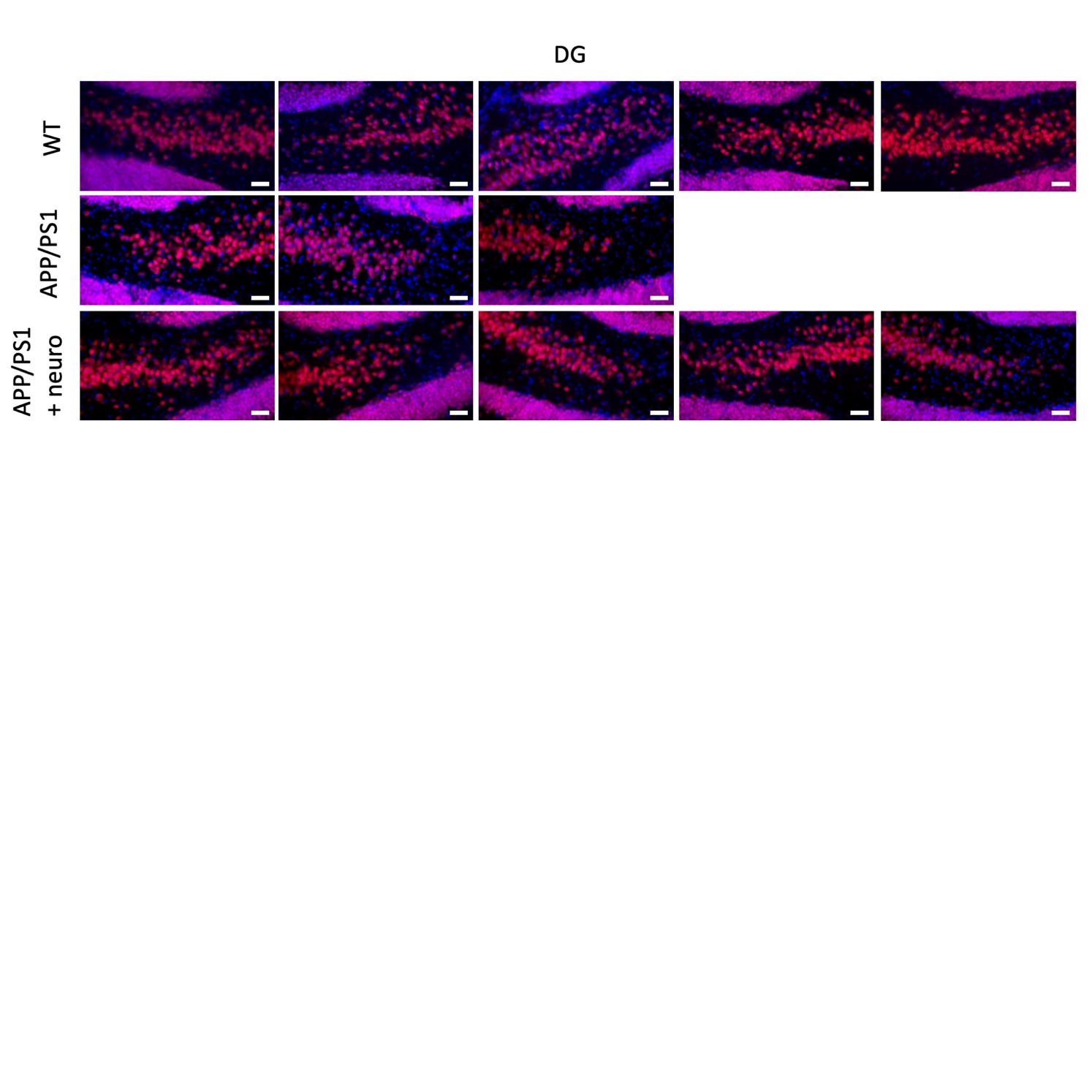


**Supplementary Figure 5.** Original immunofluorescences for Figure 3E for each 22-month-old WT and APP/PS1 animal fed with control or neurotinib diet. Coronal brain sections for NeuN marker (red) for neurons in the area of the hippocampus: CA1, CA3, and dentate gyrus (DG), and the nucleus (Hoechst staining, blue). n=3-5 animals per condition. Scale bar = 50 µm.
